# Vaccine Development against Infectious Diseases: State of the Art, New Insights, and Future Directions

**DOI:** 10.3390/vaccines11111632

**Published:** 2023-10-25

**Authors:** Fatima Conceição Silva, Paula Mello De Luca, Josué da Costa Lima-Junior

**Affiliations:** Laboratory of Immunoparasitology, Oswaldo Cruz Institute—Fiocruz, Avenida Brasil 4365-Pavilhão 26-4º andar, Rio de Janeiro 21040-900, Brazil; josue@ioc.fiocruz.br

In the 18th century, English physician Edward Jenner laid the foundation for modern vaccination by achieving protection against variola. His observations led to the development of many widely used vaccines, marking a significant milestone in medical history. A long road has been paved since then, including the announcement of global smallpox eradication through vaccination in 1980. Following this remarkable achievement in public health, the path forward has grown increasingly complex due to new regulations, evolving ethical principles, and the proliferation of misinformation. In our view, addressing these emerging demands, uncertainties, and conflicts necessitates a strong foundation in science. Globally, dedicated researchers are actively addressing these challenges, especially in the ever-evolving landscape of infectious diseases, where vaccines remain a beacon of hope, offering the promise of prevention and protection against a multitude of pathogens. 

According to the WHO, “immunization is a key component of primary health care and an indisputable human right. It is also one of the best health investments money can buy” [1]. COVID-19 vaccines have demonstrated effective immunization in controlling the pandemic, leading to a significant decrease in case numbers in the countries that achieved adequate vaccine coverage. Regrettably, these vaccines have also highlighted a new challenge confronting the scientific community: vaccine hesitancy, included in 2019 by the WHO in its list of “10 Threats to Global Health,” along with some infectious diseases such as Ebola, influenza, dengue, and HIV [2].

Presently, over 20 vaccines against infectious diseases are broadly used worldwide to prevent human infections. Although this is significant, this number becomes proportionately tiny when we think about the number of infectious diseases that currently affect humans and animals in the world, evidencing significant knowledge gaps concerning specific pathogens, immunopathology, and the intricate interplay between pathogens and hosts. Consequently, some diseases that are prevalent worldwide, even with massive attention, still do not have an effective vaccine available or even a pre-clinical or clinical trial in progress.

In this Special Issue of the *Vaccines* journal, we embark on a comprehensive journey through the realm of vaccines, exploring their pivotal role in combating infectious diseases. This Special Issue aimed to consolidate new findings and reviews from researchers in the field of vaccinology for infectious diseases, whether relating to human or veterinary vaccines. Our objective was to disseminate reliable knowledge generated using both new and traditional technologies in vaccine development, immunopathogenesis, and “in vitro” studies, as well as in experimental models and clinical trials. We are confident that the results obtained during the development of vaccines for specific infectious diseases, regardless of their stage of development, can shed light on questions that remain challenging for others. Through this endeavor, experts have delved deeply into various subjects, dissecting and exploring them. We anticipate that bringing these findings together will foster fresh insights, inspiring researchers to persevere in their efforts to create innovative vaccine solutions, including new formulations and administration routes.

The field of vaccinology continues to achieve remarkable progress in combating infectious diseases that endanger both human and animal populations. In this comprehensive topic, we focus on the latest advancements in vaccine research, highlighting significant discoveries and their implications for global health. From Toxoplasma gondii to (re)emerging diseases, such as Leishmaniasis, malaria, Leptospirosis, hepatitis B, Shigella flexneri, varicella, and yellow fever, we explore the innovative strategies employed in developing effective vaccines and comprehending their immunological mechanisms.

Regarding protozoan parasites, papers have focused on three globally prevalent parasites: Toxoplasma gondii, Leishmania spp., and Plasmodium spp. In the case of Toxoplasma gondii, co-immunization with DNA vaccines expressing the SABP1 and SAG1 proteins has demonstrated significant potential in enhancing mice’s resistance to acute Toxoplasmosis, suggesting DNA vaccines’ promise in combating this widespread parasite and protecting both humans and animals [3]. In Leishmania research, the identification of immunodominant proteins in Leishmania (Viannia) naiffi subproteome paves the way for creating a pan-specific vaccine against this debilitating disease [4]. By targeting antigens that evoke well-regulated immune responses, researchers are making strides toward an effective solution for Leishmaniasis. Additionally, exploring degron pathways in *Leishmania* spp. sheds light on how proteases influence host immune responses. This discussion delves into the potential role of proteases in guiding the host’s immune response and their relevance to vaccine development, unraveling a crucial aspect of *Leishmaniasis* pathogenesis [5]. In Malaria research, the B-Cell epitope mapping of the *Plasmodium falciparum* malaria vaccine candidate GMZ2.6c offers valuable insights into the development of immunity against this deadly disease. By identifying the key epitopes recognized by antibodies, researchers aim to bolster our understanding of protective immunity [6].

Unfortunately, many individuals are at risk of contracting multiple infectious diseases simultaneously, and the literature has shown the potential for infectious agents to interfere with the immune response during co-infection. Therefore, it is crucial to consider the concept of co-infection and comorbidity when assessing the effectiveness of vaccination. Additionally, nutritional status and access to healthcare facilities can increase the challenges in controlling infectious diseases, especially in areas of poverty. A review published on this topic discusses the state of art in schistosomiasis and malaria vaccines, two of the main infectious diseases that occur around the globe [7]. 

The description of the epitopes capable of stimulating a protective immune response has a central role in vaccine development. Identifying B-cell linear epitopes within Sphingomyelinase 2 (Sph2) represents a significant stride in the fight against leptospirosis. These epitopes, which are naturally recognized by patients infected with pathogenic *Leptospires*, hold promise for the development of multi-epitope vaccines against this neglected tropical disease [8].

Concerning vaccine design and formulations, the development of a pentavalent Shigella flexneri LPS-based vaccine candidate offers hope in the battle against Shigella infections. This study highlights the safety and immunogenicity of this vaccine, marking progress toward a multivalent solution [9]. In this connection, the immune response elicited during an in vitro model of monocyte stimulation with the BCG Moreau Vaccine is presented. The authors underscore the potential significance of biomarkers in immune stimulation and explore the role of the innate immune response in vaccine development and design [10].

Virus infections pose a significant global public health challenge due to their rapid spread among diverse populations and the increased risks for vulnerable individuals. The absence of effective drugs for containment has thrust vaccines into the spotlight. In this context, the evaluation of two adjuvant formulations for an inactivated yellow fever 17DD vaccine candidate demonstrates the potential of non-live vaccine alternatives. These formulations exhibit promising immunogenicity, underscoring the possibility for safer vaccines against this deadly virus [11]. Regarding virus infection, the present Special Issue published three papers with valuable information about virus circulation in different countries: Italy, Taiwan, and India. Valuable data on the effectiveness of vaccination programs are reported in college students after vaccination in Taiwan. The authors underscore the importance of vaccination in reducing the prevalence of this viral infection among young adults [12]. Moreover, a sero-epidemiological study of varicella in the Italian general population sheds light on the impact of vaccination. With increasing seroprevalence data, this study contributes to our understanding of the disease’s dynamics post-vaccination [13]. In the context of the Chikungunya virus, one article discusses the outbreak history of CHIKV in India and emphasizes the subsequent necessity to advance knowledge for effective treatment and preventive strategies [14].

The literature within the field of vaccinology is becoming increasingly intricate. In such a complex landscape, the value of comprehensive reviews to organize and structure this wealth of information cannot be overstated. Consequently, review papers presented and discussed different aspects of vaccine development. One notable approach is the use of antibiotic-free nanoplasmids in DNA vaccines, which help mitigate the concerns related to antibiotic resistance genes. These nanoplasmids exhibit comparable transfection efficiency to conventional plasmids, making them a viable option for subunit vaccine development [15]. Insights to expedite the process of vaccine development are also discussed, aiming to offer a potential guide to produce new vaccines [16]. Lastly, the intricate landscape of vaccination during pregnancy is discussed. By surveying vaccination decisions and safety considerations, we gain insights into the challenges and opportunities of immunization in expectant mothers, particularly in the context of emerging diseases like COVID-19 [17].

In the unwavering commitment to safeguarding public health, vaccine research continues to advance, offering hope in the battle against challenging infectious diseases. The collective efforts of scientists worldwide have illuminated novel pathways to immunity and underscored the critical importance of vaccination. In a world where emerging diseases pose constant threats, these advancements in vaccinology serve as signs of progress. With every breakthrough, we edge nearer to a future in which effective vaccines protect us against a myriad of infectious foes, ensuring the well-being of generations to come.

In conclusion, this Special Issue, comprising a diverse array of research and review articles, reflects the dynamism and innovation within the field of vaccine research. As we navigate the complex landscape of infectious diseases, vaccines remain our steadfast allies in the quest for global health security. Featuring a total of 12 research and 3 review articles, this Special Issue underscores the profound impact of vaccines on public health. We hope the readers will both enjoy and benefit from the information presented in this research topic. We also extend our heartfelt appreciation to the authors for their exceptional contributions. Just keep going: vaccines are necessary and we still have a lot of research to carry out. 
